# A Review of the Activity Regulation of Au and Pt Bimetallic Nanozymes and Their Application in Food Safety Analysis

**DOI:** 10.3390/bios16060325

**Published:** 2026-06-03

**Authors:** Zhengxin Zhou, Muci Wu, Rui Zhang, Wangting Zhou, Jiaojiao Zhou, Jingren He

**Affiliations:** National R&D Center for Se-rich Agricultural Products Processing, Hubei Engineering Research Center for Deep Processing of Green Se-rich Agricultural Products, School of Modern Industry for Selenium Science and Engineering, Wuhan Polytechnic University, Wuhan 430023, China; 20241411011@whpu.edu.cn (Z.Z.); muciwu@whpu.edu.cn (M.W.); rui.zhang@whpu.edu.cn (R.Z.); zhouwangting01@whpu.edu.cn (W.Z.)

**Keywords:** AuPt bimetallic nanozyme, regulation of catalytic activity, food safety analysis, biosensor

## Abstract

Food safety problems caused by pesticide residues, heavy metals, foodborne pathogens, mycotoxins and other hazards seriously threaten public health. Traditional detection methods have the limitations of cumbersome operation, high cost and poor stability, which make it difficult to meet the needs of rapid and sensitive detection on site. As a new material, nanozymes have the advantages of high stability, low cost and high catalytic activity, showing great application potential in food safety analysis. Among them, gold–platinum (AuPt) bimetallic nanozymes have attracted much attention due to their synergistic catalytic effect, good biocompatibility and modifiability. In this paper, the synthesis methods of AuPt bimetallic nanozymes were systematically reviewed, including chemical reduction, sol–gel, microemulsion, electrochemical deposition, and so on. The control effect of AuPt bimetallic nanozymes on catalytic activity was discussed from the aspects of composition, morphology, structure, external environment and composites with other nanomaterials. The research progress of AuPt bimetallic nanozymes in the detection of pesticide and veterinary drug residues, heavy metal ions, mycotoxins, foodborne pathogens, food additives and food freshness was introduced. Finally, the challenges and future development of AuPt bimetallic nanozymes in food safety analysis were prospected, aiming to provide theoretical reference and design ideas for the construction of a high-performance food safety rapid detection platform.

## 1. Introduction

Food safety issues, such as pesticide residues and foodborne pathogenic bacteria contamination, may also lead to acute poisoning or long-term health problems [1] It is estimated that the global population will reach 9 billion by 2050, requiring a 70% increase in food supply [2]. Meanwhile, as food trade becomes increasingly globalized, environmental pollution risks have grown more widespread and harder to control. It is estimated that 600 million people worldwide suffer from foodborne diseases caused by food hazards each year. This staggering figure underscores the critical need for heightened attention to food safety issues. Ensuring the safety of food products is essential not only for protecting public health but also for enhancing consumers’ confidence in the food supply [3]. Food hazards present a significant threat to public health and pose a major challenge on a global scale. Efforts to address food safety often focus on increasing consumers’ understanding of food safety and improving regulatory measures. Nevertheless, issues such as pesticide residues, heavy metals, foodborne pathogens, toxins, and food additives continue to represent a considerable risk to public health. These challenges not only jeopardize the well-being of consumers but also threaten the economic interests of businesses within the food industry. Therefore, people urgently need timely and accurate detection methods. Over the past few decades, various detection techniques have been employed to detect food contaminants and ensure food quality and safety. Among them, high-performance liquid chromatography (HPLC), polymerase chain reaction (PCR) and enzyme-linked immunosorbent assay (ELISA) have been widely used because of their high detection accuracy. Chromatographic-based detection methods (i.e., HPLC and LC-MS) display high detection accuracy and sensitivity, but they usually rely on complex instruments and large quantities of sample preparation [4]. PCR has been widely used in the detection of foodborne pathogens, but its complex primer design and sensitivity to false-positive results may limit its wider application [5]. Furthermore, owing to its high specificity, ELISA is also widely used in food safety detection, but the relatively high cost of enzymes and the complex preparation of antibodies may hinder its application [6].

In order to address these challenges, researchers have in recent years focused on nanozymes. By synthesizing nanomaterials with catalytic activity similar to that of natural enzymes, they aim to develop a novel detection strategy for food safety analysis with high efficiency, stability, simplicity and scalability [7].

Nanozymes refer to inorganic or organic nanomaterials with enzyme-like catalytic activities (e.g., peroxidase, oxidase, superoxide dismutase, etc.) [8]. Compared with natural enzymes, nanozymes have obvious advantages in terms of stability, yield and tolerance to different operating environments. Firstly, most nanomaterials possess better thermal and chemical stability and can maintain catalytic activity in harsh or variable environments. Secondly, they can often be prepared on a large scale at a relatively low cost. In addition, nanozymes can achieve the required catalytic specificity or multifunction by adjusting their composition, morphology, size and surface modification [9]. Nanozymes are usually designed to be combined with corresponding signal response modes (such as colorimetry, electrochemistry, fluorescence, surface enhanced Raman scattering, etc.), so as to achieve rapid and sensitive target detection. They show great potential in the fields of food safety analysis [10], environmental monitoring [11], and biosensors [12], among others.

**Figure 1:**
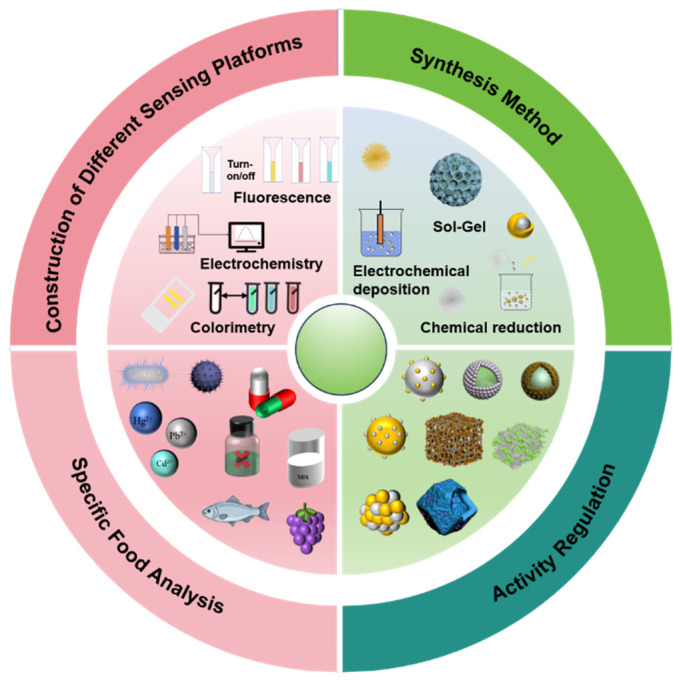
Scheme of the activity regulation of Au and Pt bimetallic nanozymes and their application in food safety analysis.

Among the many nanozyme-based systems, gold–platinum bimetallic nanozymes have attracted much attention. Gold (Au) and platinum (Pt) are widely used noble metal nanomaterials with different surface electronic structures and catalytic properties. Combining the two in the same nanocatalyst can not only achieve synergistic effects between the two metals but also further regulate the catalytic activity and stability by changing the alloy composition and structure [13,14]. AuPt nanozymes generally possess the following advantages (Figure 1): (1) Multiple enzyme-like activities, including peroxidase-, oxidase-, or other enzyme-like activities simultaneously. (2) Good biocompatibility and low toxicity. Noble-metal-based nanomaterials possess the merits of high stability, low toxicity, and good biocompatibility [15]. AuPt-based nanozymes exhibit better catalytic stability than natural enzymes and are suitable for on-site detection and analysis in complex food matrices [16]. (3) Flexible modifiability. The surface of AuPt nanozymes can be modified with various molecules (e.g., antibodies, oligonucleotides, and polymers) to achieve targeted recognition, signal amplification, or specific environmental responses [17]. These advantages lay a theoretical and methodological foundation for the applications of AuPt-based nanozymes in food safety analysis.

Although there have been many reviews in recent years that systematically summarize the classification, catalytic mechanisms, and applications of nanozymes in biomedical or environmental monitoring, some of the literature has also explored the preliminary sensing strategies of conventional nanozymes in the field of food safety. However, most existing reviews focus on a wide range of nanomaterials or a single detection mode, and there are few studies specifically analyzing the precise regulation mechanism of the catalytic activity of Au and Pt, two high-performance precious metals, and their intrinsic relationship with multimodal analysis applications in food. Unlike previous reviews, this article systematically outlines how to enhance the catalytic performance of Au and Pt bimetallic nanozymes in multiple dimensions through composition ratios, defect engineering, and composites with emerging nanomaterials such as carbon dots and metal–organic frameworks (MOFs). On this basis, the latest progress of Au and Pt bimetallic nanozymes in the detection of agricultural and veterinary drug residues, heavy metals, foodborne pathogens, and food freshness, from single signal output to multimodal and intelligent, has been comprehensively summarized. This review aims to provide clear theoretical guidance and a forward-looking perspective for breaking through the interference of complex food matrices and designing the next generation of highly sensitive on-site real-time detection platforms.

## 2. Synthesis of AuPt-Based Nanozymes

The synthesis methods of AuPt-based nanozymes are diverse, flexible, and controllable, which lays the foundation for investigating their specific catalytic performance and structure–activity relationship. According to the differences in synthetic parameters such as metal ions, reducing agents, solvents, ligands, temperature, pressure, and reaction time, these methods can be mainly classified into the following categories.

### 2.1. Chemical Reduction Method

Chemical reduction is one of the most extensive and economical methods to prepare AuPt nano-alloys and core–shell structures with controllable size, morphology and composition [18]. In the synthesis process, Au and Pt precursors, such as HAuCl_4_, H_2_PtCl_6_ or K_2_PtCl_4_, are reduced by appropriate reductants in the presence of solvents, stabilizers or surface ligands. The formation of AuPt nanoparticles generally includes metal ion reduction, atomic nucleation and particle growth. In this process, the final particle size, morphology and metal distribution are determined by the reduction rate, precursor ratio, reaction temperature, pH value and ligand coordination [19,20].

**Figure 2:**
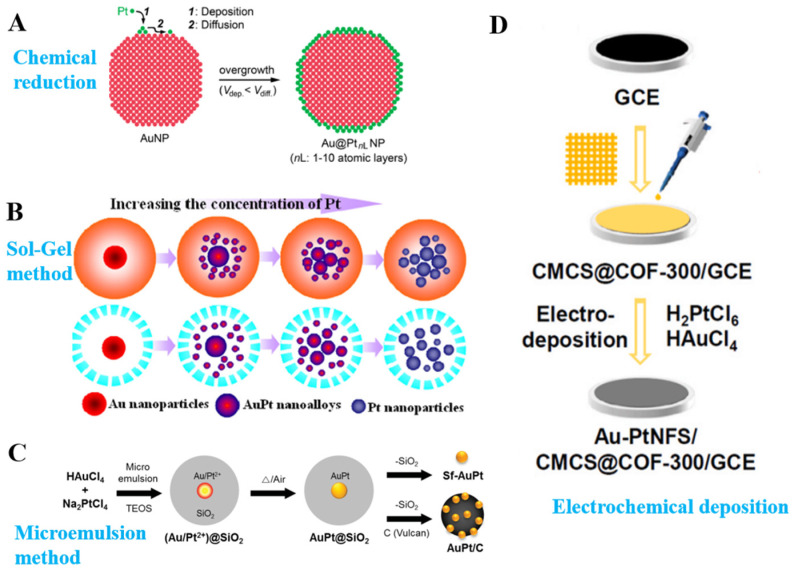
Scheme of different synthesis methods for AuPt nanozymes. (**A**) Chemical reduction. Adapted with permission from Ref. [24], Copyright (2017) American Chemical Society. (**B**) Sol–Gel method. Adapted with permission from Ref. [27], Copyright (2016) Elsevier. (**C**) Microemulsion method. Adapted with permission from Ref. [31], Copyright (2015) Wiley. (**D**) Electrochemical deposition. Adapted with permission from Ref. [33], Copyright (2025) Elsevier.

Chemical reduction can be divided into co-reduction and sequential reduction according to the reduction order. During the co-reduction process, Au and Pt precursors are introduced into the same reaction system and reduced simultaneously or almost simultaneously. Due to the high reduction potential of Au ions, Au is usually reduced first, and then Pt is reduced and deposited to form alloyed or partially alloyed AuPt nanostructures [21,22]. In the sequential reduction route, one metal is first reduced to seed, and then another metal is deposited on the seed surface. This strategy is usually used to construct core–shell structures [23]. For example, Gao et al. prepared Pt-decorated Au nanoparticles (Au@Pt NPs) with shell thickness and alloy uniformity through the seed-mediated method (Figure 2A) [24]. Owing to the dual functionalities, plasmonics and catalysis, the prepared Au@Pt NPs could enhance detection sensitivity by two orders of magnitude in the lateral flow assays.

Ligands and stabilizers also play important roles in chemical reduction. Common ligands, such as citrate, PVP, CTAB and sulfhydryl-containing molecules, can be adsorbed on the surface of nanoparticles or coordinated with metal ions to inhibit aggregation and regulate crystal growth [25,26]. Their selective adsorption on different crystal planes further affects the morphology of nanoparticles and exposed active centers. In addition, functional ligands can improve the dispersion, stability and surface modification ability of nanoparticles. Therefore, chemical reduction provides a flexible method for the preparation of AuPt nanostructures, and the catalytic performance of the materials can be optimized by adjusting the reduction kinetics, the composition of metal precursors and the surface ligand environment.

### 2.2. Sol–Gel Method

This method involves embedding prefabricated Au, Pt, or AuPt nanocrystals into a silica (or ordered mesoporous) system synthesized via the sol–gel process. The template is then removed by carbon/resin templating or organic calcination to obtain small nanocores or alloy particles dispersed in a hollow/multi-core confined space. The limited cavities and ordered channels inhibit particle agglomeration, increase the effective surface area, and enhance mass transport, which favors the diffusion of substrates or products. The alloying degree and particle size can be controlled by optimizing the precursor ratio and calcination temperature. Meanwhile, the SiO_2_ support shell also serves a protective and stabilizing function.

Qi et al. utilized an RF-COOH (2,4-dihydroxybenzoic acid formaldehyde) resin template combined with a one-step sol–gel method to embed Au, Pt, or AuPt into an ordered radially mesoporous SiO_2_ shell (Figure 2B) [27]. After calcination, hollow SiO_2_ was obtained, containing dispersed AuPt nanocrystals with sizes ranging from 2 to 6 nm. These catalysts exhibited excellent catalytic performance toward the epoxidation of styrene. This route enables the fabrication of highly dispersed, small-sized, and controllably alloyed nanozyme catalytic sites within confined spaces, which is beneficial for suppressing sintering and improving cycling stability. However, the process involves multiple steps and is limited by the scalability of the template. If a bare metal surface is required, shell removal is necessary, which may introduce corrosion or loss of material.

### 2.3. Microemulsion Method

This method forms nanoscale reaction microcavities in an oil/water/surfactant system, thereby confining the nucleation and growth of nanoparticles within these microcavities, and ultimately yields uniform and controllable AuPt nanoparticles. The obtained structures include common core–shell, alloy, and special intermediate structures [28]. This method has the advantages of precise nanosize control, high product purity, and mild reaction conditions. It is suitable for the preparation of uniformly sized, morphologically specific AuPt nanozymes (such as nanocubes and nanodisks). Their catalytic activity can be tuned by adjusting the microcavity size, surfactant type, precursor concentration, and addition method. The composition of the solvent and microemulsion can affect the coordination state and formation kinetics of the precursors, thereby influencing the occurrence of surface enrichment or monatomic layer deposition [29,30]. Systematic studies by both Kim et al. [31] and Landry et al. [32] demonstrate that by using microemulsions and controlling the precursor addition temperature, Pt^2+^ or Pt^2+^-like deposits can form monolayers or thin films on the surface of Au nanocrystals (Figure 2C). These layers can subsequently be transformed into alloys via solid-state reactions or heat treatment. This approach enables the formation of surface-modifying thin layers (such as those derived from Pt^2+^), providing precursor structures for subsequent alloying or core–shell construction.

### 2.4. Electrochemical Deposition Method

Au and Pt atoms are deposited onto conductive substrates (such as Au, Pt, graphite, etc.) or nanostructures via electrochemical redox reactions to form alloy layers or nanostructures (Figure 2D) [33]. This method allows the preparation of uniform, large-area nanozyme films, which can be easily integrated with electrochemical detection devices. Electrodeposition can be directly constructed on the electrode without further loading, facilitating its coupling with electrochemical tests and applications. However, this method is limited in achieving large-scale dispersed loading, and attention should be paid to deposition uniformity and surface cleanliness.

The synthesis method depends on the target size, morphology, composition, surface properties, and subsequent application requirements of the AuPt nanozyme. For example, materials requiring high catalytic activity may favor the formation of uniform alloy structures, whereas for biosensing applications, specific functional groups may be required on the surface to conjugate recognition molecules. The advantages and disadvantages of different synthesis methods are shown in Table 1.

## 3. Regulation of Catalytic Activity of AuPt-Based Nanozymes

The catalytic activity of AuPt nanozymes is not fixed; it can be precisely regulated via various strategies to meet the requirements of different detection scenarios. Their activities are mainly determined by intrinsic material properties (composition, structure, and morphology) and external environmental factors.

### 3.1. Regulation of Composition and Atomic Ratio

The atomic ratio of Au to Pt is a key factor affecting its catalytic activity. Pt exhibits stronger redox capability than Au, and an appropriate amount of Pt can significantly enhance the enzyme-like activity of Au, especially its peroxidase-like activity. For various substrates, AuPt nanozymes with different atomic ratios display distinct catalytic activities. Chen et al. [34] systematically evaluated the enzyme-like catalytic efficiency of AuPt with different bimetallic compositions, and Au:Pt at 3:1 exhibited the highest activity toward glucose oxidation in solution. Moreover, cell co-cultured AuPt nanoparticles showed favorable biological performance for glucose sensing. Liu et al. found that in the AuPt alloy, Au plays the role of glucose oxidase, while Pt plays the role of peroxidase [35]. The activity of glucose oxidase is the highest in Au_15_Pt_5_, about 240%, which is significantly higher than that of pure Au (90%), Au_13_Pt_7_ (100%), Au_10_Pt_10_ (25%) and pure Pt (10%). The peroxidase-like activity of pure Pt was about 140%, while that of Au_13_Pt_7_ and Au_10_Pt_10_ was about 100% and 90%, respectively. Unfortunately, the peroxidase-like activity of pure Au and Au_15_Pt_5_ was only about 5%. The cascade catalytic activity of the materials was further investigated. Compared with Au_15_Pt_5_, Au_10_Pt_10_, pure Au or pure Pt nanoparticles, the highest activity was obtained at a ratio of Au to Pt of 13:7.

### 3.2. Regulation of the Shape of Nanoparticles

The shape of nanoparticles (such as spheres, cubes, nanorods, nanowires, etc.) affects their specific surface area, surface atomic exposure sites and electronic structure, and then affects their catalytic activity [36]. For example, cubic nanostructures typically expose more crystal facets, and Pt exhibits superior catalytic activity on certain crystal facets. Therefore, the oxidase-like activity of AuPt cubic nanozymes may be higher than that of spherical ones. Meanwhile, the size of nanoparticles also affects their catalytic activity. Smaller size generally provides a larger specific surface area, which favors catalytic reactions, although activity may also be altered due to the quantum size effect.

In addition to the control of composition and morphology, crystal surface engineering, defect engineering, monatomic site and interface electron transfer are also used to control the catalytic activity of AuPt bimetallic nanozymes. Crystal plane engineering can selectively expose high-energy crystal planes with different atomic arrangements, so that the material can change the adsorption and activation of H_2_O_2_, O_2_, TMB and other reaction substrates [37]. Defect engineering, including vacancies, lattice distortions, edges, steps and twin boundaries, can generate more coordination unsaturated sites and accelerate charge transfer in catalytic reactions [38]. At the same time, the Au or Pt sites dispersed by atoms can maximize the utilization of metal atoms and provide clear catalytic centers [39,40].

The interfacial electron interactions between Au and Pt, or between metal nanoparticles and MOFs, carbon materials and oxides can further regulate the d-band centers, surface valence states and electron transfer pathways, thereby improving the activities of peroxidase, oxidase or peroxidase [34,41]. For example, Wang et al. synthesized AuPt spiny nanotubes (AuPt SNTs) through a one-pot synthesis method, which has rich defect sites and lattice strain surfaces and synergistically enhanced the ethanol oxidation reaction (EOR) performance [42]. The unique ridge-like nanotube structure, coupled with the surface and lattice strains with abundant defects, provides an optimized electronic structure, enhances the exposure of active sites, and improves the kinetics of reactant diffusion.

### 3.3. Regulation of External Environmental Factors

In practical applications, the catalytic activity of nanozymes is significantly influenced by the conditions of the reaction medium. For instance, pH is the key factor affecting the activity of nanozymes. Most nanozymes show high reaction rates under neutral or weak acid conditions [43]. Temperature not only changes the kinetic rate of reaction but also affects the catalytic activities of nanozymes. There is usually an optimal temperature range for nanozymes. Beyond this range, the activity may be decreased or inactivated [44]. At the same time, surface modification strategies such as polymer coating, ligand coordination or assembly into composite materials can enhance the thermal stability and durability of nanozymes [45]. The catalytic rate increases with the rise in substrate concentration and eventually approaches saturation, exhibiting kinetics similar to Michaelis–Menten [46]. To improve the anti-interference ability, targeted design can be employed in material selection and surface functionalization, or appropriate sample pretreatment methods can be used to remove or buffer interferents. In conclusion, to achieve stable, sensitive and reproducible detection using nanozymes in complex food matrices, systematic process optimization should be implemented, including buffer system and pH control, temperature and reaction time optimization, kinetic characterization, anti-interference design, and synergistic strategies.

### 3.4. Combination with Other Nanomaterials

To meet the requirements of high sensitivity, high selectivity, multi-target detection and signal enhancement in food analysis, researchers have explored strategies for integrating AuPt nanozymes with other nanomaterials. Such synergistic effects take advantage of the unique merits of different nanomaterials to compensate for their individual performance deficiencies, thereby constructing catalytic platforms with more comprehensive functions and superior performance, and extending their application potential in complex food matrices.

As a new “zero-dimensional” carbon nanomaterial, electron donor and acceptor functional groups on CDs can undergo interfacial coupling with Au/Pt surfaces, leading to changes in the metal surface valence states (e.g., Pt^2+^/Pt^0^ and Au^4+^). This alters the adsorption energy of substrates and the reaction energy barrier, ultimately enhancing or regulating the rates of peroxidase-like and oxygen reduction reactions. Liu et al. [47] found that both hydroxyl and carbonyl groups of CDs can coordinate with Pt^2+^ and then adjust the charge state of Pt nanozymes to promote Pt@CD formation and improvement their POD-like activity. Similarly, Tran et al. [48] successfully combined AuPt bimetallic nanocubes with graphene quantum dots using a simple and easy method. The results indicate that this nanocomposite exhibits high catalytic activity for glucose detection from 1 pM to 1 M.

The combination of AuPt nanozymes and metal–organic frameworks (MOFs) aims to optimize their catalytic performance by utilizing the highly tunable structural properties of MOFs. MOFs not only serve as ideal carriers, but their unique microenvironment can also endow nanozymes with higher catalytic efficiency and selectivity [49]. The highly ordered pore structure of MOFs provides an ordered growth space for AuPt nanozymes, effectively preventing nanoparticle aggregation and optimizing substrate diffusion pathways [50]. For example, the AuPt/ZIF-8-rGO composite material exhibits significantly enhanced peroxidase-like activity due to its ultra-small size and high dispersibility of nanoparticles, unique sandwich porous structure, and strong metal–carrier interaction. The electrochemical sensor constructed based on this material can achieve high sensitivity and wideband detection of H_2_O_2_, with a linear range of 100 nM to 18 mM and a detection limit as low as 19 nM [41].

In general, the regulation of catalytic activity of AuPt nanozymes mainly depends on the active site exposure, electronic structure regulation, substrate adsorption behavior and interfacial electron transfer process. Pt-rich sites are usually used as the main redox active centers, which can promote the activation of H_2_O_2_, O_2_ and chromogenic substrates. Au has an important effect on the overall catalytic performance by adjusting the local electronic structure around Pt, thus enhancing the structural stability and optimizing the adsorption behavior of reaction intermediates. Therefore, AuPt nanozymes usually need an appropriate Au/Pt atomic ratio to achieve a balance between the number of Pt active sites, the AuPt electronic coupling effect and the structural stability of nanoparticles.

From the perspective of the structure–activity relationship, component regulation mainly affects the electronic environment and active site density. The specific surface area, crystal surface exposure and mass transfer efficiency are determined by morphology and size control. Defect engineering and crystal plane engineering can introduce more low coordination atoms to promote substrate adsorption and activation. Carrier loading and heterostructure construction are helpful to improve the dispersion of nanoparticles and strengthen interfacial electron transfer. A comparison of representative kinetic parameters of Au and Pt bimetallic nanozymes is shown in Table 2.

## 4. Specific Food Analysis

### 4.1. Establishment of Sensing Mechanism

The most common analysis strategy of AuPt nanozymes in food safety analysis is catalytic color amplification detection, which uses the activity of AuPt peroxidase-like enzyme or oxidase-like enzyme to catalyze the color change of substrates such as TMB, ABTS and AEC, so as to realize the rapid detection of veterinary drug residues, pesticides, histamine, food additives and other target substances [56,57,58]. In addition, some of the target compounds can directly regulate the catalytic activity of AuPt nanozymes, forming activity inhibition or recovery sensing mode. Heavy metal ions, reducing small molecules or antioxidants can affect the surface active sites of nanozymes through adsorption, coordination or redox reaction, and then change the color signal intensity [55,59]. At the same time, in order to improve the specificity of detection, AuPt nanozymes can be combined with antibodies, aptamers, molecularly imprinted polymers, antibiotic recognition units or magnetic materials to construct a specific recognition system. In the detection of small molecular pollutants, the competitive immune model is often used; in the detection of foodborne pathogens, the sandwich recognition structure and magnetic separation enrichment strategy are often combined [60,61,62]. At this time, AuPt is mainly responsible for catalytic amplification and signal output.

### 4.2. Detection of Hazardous Substances: Drug Residues, Heavy Metal Ions and Mycotoxins

#### 4.2.1. Drug Residues

Drug residues (such as veterinary drugs and pesticides) pose a serious threat to human health. Veterinary drug residues can directly cause side effects such as allergy, poisoning, carcinogenesis and teratogenicity [63]. The abuse of antibiotics also leads to the spread of drug-resistant strains through the food chain, which may lead to superbacterial infection and make traditional antibiotic treatment ineffective [64,65]. Pesticide residues can cause chronic poisoning, damage the nervous, endocrine and immune systems, and even affect children’s nerve development [66]. Therefore, the development of innovative detection technology is essential to ensure food safety [67].

**Figure 3:**
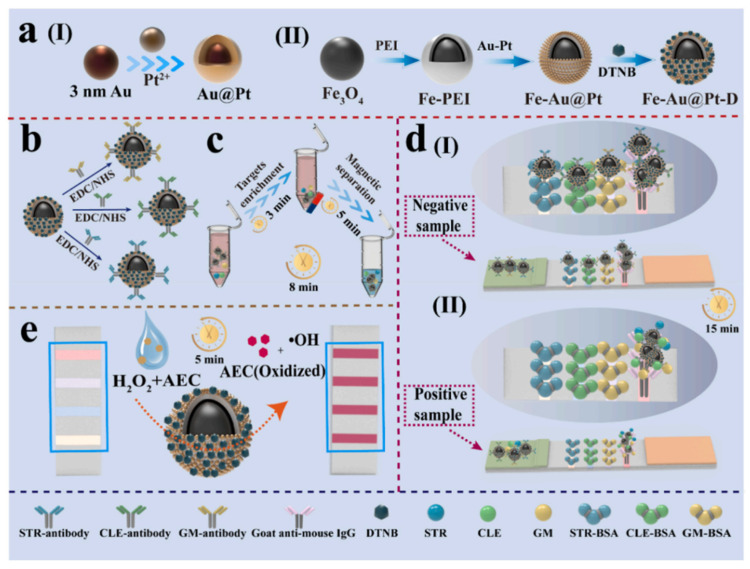
Scheme of a lateral flow immunoassay for the colorimetric detection of multiple drug residues based on Fe–Au@Pt core–satellite nanozymes. (**a**) synthesis of Au@Pt NPs (**I**) and Fe–Au@Pt core–satellite NPs (**II**); (**b**) preparation of three kinds of immuno–Fe–Au@Pt magnetic nanozymes; and (**c**) simultaneous magnetic enrichment of STR, CLE, and GM from sample solutions and (**d**) their detection on Fe–Au@Pt-based multiplex LFA. (**e**) Catalytic mechanism of the Fe–Au@Pt nanozyme on LFA strips. Reprinted with permission from Ref. [68]. Copyright (2024) Elsevier.

Researchers have developed different AuPt-nanozyme-based immunochromatography (LFIA) strategies to address residues of three veterinary drugs: gentamicin (GM), clenbuterol (CLE), and streptomycin (STR). For example, Xu et al. designed a core–shell-structured nanozyme (Fe–Au@Pt–LFA) with magnetic Fe_3_O_4_ as the core and Au/AuPt bimetallic as the satellite layer [68] (Figure 3). In the LFIA sensing mode, the composite system first utilizes a magnetic core to achieve rapid pre-enrichment of targets in complex matrices. Then, through the extremely high peroxidase-like activity of the AuPt satellite layer, it efficiently catalyzes the substrate 3-amino-9-ethylcarbazole (AEC) to generate insoluble red precipitates, converting microscopic immune competition binding into macroscopic rapidly amplified colorimetric signals. This enrichment catalysis dual signal amplification strategy reduces the detection limits (LODs) of GM, CLE, and STR to 10.1, 6.3, and 1.1 pg/mL, respectively. On the same detection target, Zheng et al. constructed a three-dimensional multilayer sheet-like nanozyme (GO/Au AuPt) using graphene oxide (GO) as a carrier [69]. This study uses flexible and high-specific-surface-area GO as the substrate and sequentially loads 20 nm AuNPs and tens of thousands of densely distributed 5 nm AuPt nanosatellite particles through electrostatic self-assembly. This 3D layered structure greatly increases the reaction contact area and optimizes electron transfer. In the catalytic AEC deposition mode, the detection limits for RAC, CLE, and GM reached 2.61, 3.6, and 4.9 pg/mL, respectively, and the dynamic linear detection range was expanded by nearly a hundred times (spanning from pg/mL to ng/mL levels). Both studies have demonstrated that the AuPt bimetallic nanozyme system improves sensitivity.

AuPt nanozymes also confer a distinct advantage in signal amplification for the determination of organophosphorus pesticide residues. Chen et al. established a bio-barcode immunoassay mediated by Au@Pt nanozymes, which used complementary single-stranded DNA labeled with Au@Pt nanozymes as signal tags [70]. Taking advantage of the high catalytic activity of Au@Pt nanozymes, the method achieved efficient amplification of fluorescent signals without the involvement of natural enzymes. While simplifying the detection process, it realized the trace detection of chlorpyrifos, triazophos and parathion, with limits of detection as low as 1.47 to 9.88 ng/kg, and could simultaneously complete the quantitative analysis of multiple pesticides.

In terms of innovation in detection mode, Zhang et al. prepared signal probes with Pt@Au nanozymes as the catalytic core and constructed a competitive immunoassay system combined with magnetic capture probes [57]. Relying on the high catalytic chromogenic activity of Pt@Au nanozymes toward tetramethylbenzidine, the visual and quantitative detection of omethoate could be accomplished by coupling with a smartphone, with a linear range from 0.5 to 50 μg/L and a limit of detection of 0.01 μg/L. This method featured simple operation and high specificity and was suitable for on-site rapid detection.

#### 4.2.2. Heavy Metal Ions

The presence of heavy metals in food poses a serious threat to human health and the ecological environment, such as lead ions (Pb^2+^), cadmium ions (Cd^2+^), mercury ions (Hg^2+^), and chromium (Cr^6+^). Even at trace levels, their high toxicity and bioaccumulation have attracted widespread attention [71]. At the same time, the food matrix is complex, there are many kinds of heavy metals, and they often exist in trace amounts, which brings challenges to the rapid and accurate detection and traceability. Until now, researchers have developed new detection technologies, such as nanozyme-based sensors, to achieve rapid, accurate and high-throughput detection of heavy metals [72,73].

AuPt bimetallic nanozymes can achieve label-free and highly sensitive detection through specific physical and chemical interactions with specific metal ions. For example, Zhou et al. confined AuPt bimetallic nanozymes in the mesoporous channels of branched silica nanospheres (DSNs) [74]. The dendrimer porous structure of DSNs makes the loaded AuPt nanoparticles highly dispersed and fully exposed, thus exhibiting superior peroxidase-like activity. Based on the specific interaction between Hg^2+^ and Pt (amalgam formation), the catalytic activity of AuPt@DSN was inhibited upon addition of Hg^2+^. The linear range of detection for Hg^2+^ is from 0.1 nM to 10 μM, and the detection limit is as low as 8.58 pM. 

**Figure 4:**
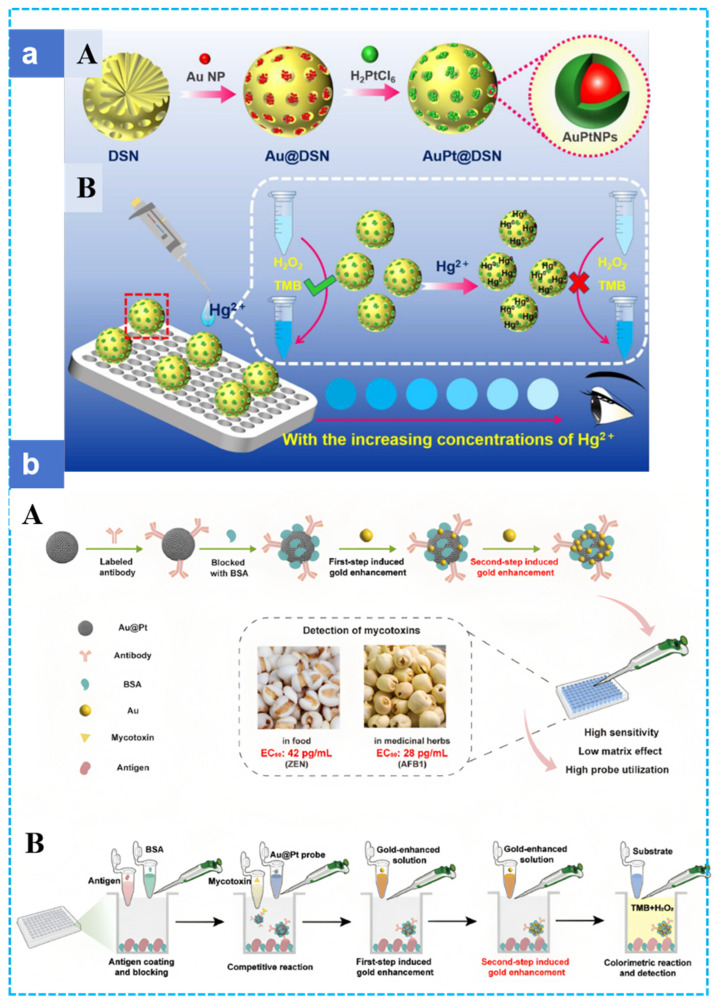
Schematic diagram of AuPt nanozyme sensor for detecting heavy metal ions and mycotoxins. (**a**) Scheme of the colorimetric detection of Hg^2+^ using AuPt@DSN nanozymes. (**A**) Preparation Procedures of AuPt@DSN and (**B**) Label-Free Colorimetric Process for Hg^2+^ Detection Using AuPt@DSN Nanozymes.Reprinted with permission from Ref. [74], Copyright (2022) American Chemical Society. (**b**) Scheme of an activity–specificity-balanced Au@Pt nanozyme immunoassay via dual-Au deposition for enhanced myco-toxin detection. (**A**) Design and construction of the Au@Pt/Au-ELISA based on the two-step gold enhancement process. (**B**) Detection workflow illustrating the specific recognition and sensitive quantification of mycotoxins using the two-step gold enhancement induced Au@Pt/Au-ELISA. Reprinted with permission from Ref. [80], Copyright (2026) Elsevier.

In addition, Zhou et al. developed a core–shell Au@Pt-nanozyme-based colorimetric assay for the detection of Cr^6+^ [75] (Figure 4a). Specifically, Au@Pt nanozymes can catalyze the oxidation of TMB into oxTMB, accompanied by a change in color. Upon the addition of Cr^6+^, oxTMB is reduced into colorless TMB, resulting in a decrease in absorbance at 652 nm. By optimizing the size of the Au core (15 nm) and the thickness of the Pt shell, the best catalytic activity and detection sensitivity were obtained. The detection limit of Au@Pt-nanozyme-based assay for Cr^6+^ is 0.5 ppb, and the linear range is from 5 ppb to 2 ppm.

In practical applications, the coexistence of multiple heavy metals is common. Therefore, it is necessary to develop a sensor system that can sensitively detect multiple heavy metals simultaneously. To meet this demand, Wu et al. designed a sensor array composed of three nanozymes: Fe-N-C, AuPt@N-C, and AuPt@Fe-N-C [76]. Different heavy metal ions have different fingerprint responses to the catalytic activities of the three enzymes. Combined with linear discriminant analysis and smartphone-based RGB mode color reading, it can distinguish Hg^2+^, Pb^2+^, Co^2+^, Cr^6+^, and Fe^3+^ ions at the same time within 5 min, and the minimum resolution concentration is 0.5 μM. It has been successfully used in the detection of seawater and salmon samples. This work provides a new strategy for high-throughput screening of heavy metal ions in complex systems.

#### 4.2.3. Mycotoxins

Mycotoxins are secondary metabolites produced by some filamentous fungi and widely exist in various agricultural products, food and feed [77]. These mycotoxins are not only highly toxic but also thermally stable and difficult to remove by conventional cooking and food processing, which poses a serious threat to human and animal health [78]. Many traditional detection methods are time-consuming and complicated, meaning they cannot meet the demand for real-time or rapid detection of mycotoxins. It is urgent to develop more efficient, sensitive, convenient and low-cost mycotoxin detection methods to protect human health [79].

The researchers prepared Au@Pt/Au composite nanozymes; the system of Au@Pt dispersed secondary Au layers was grown outside the core–shell structure [80]. This unique design not only provides sufficient Au-S bond anchor sites for the immobilization of antibody molecules to ensure the targeting specificity, but also perfectly retains the strong catalytic activity of the bare Pt shell. After the probe was integrated into the enzyme-linked immunosorbent assay (ELISA) platform, the catalytic amplification signal of *aflatoxin* B1 (AFB1) showed an exponential increase; the detection linear range was from 0.01 to 1 ng/mL (Figure 4b).

In the research of intelligent portable detection, for the detection of *zearalenone* (ZEN), researchers encapsulated AuPt nanozymes in situ in the hydrogel network crosslinked by polyionic liquid (AuPt@PIL-gel) [58]. A smartphone-assisted colorimetric sensing platform was constructed. The PIL hydrogel network can not only protect and prevent agglomeration of AuPt nanozymes but also produce specific preconcentration of toxin molecules in complex samples. Combined with the competitive immune binding mechanism, the enriched and amplified AuPt catalytic color signal is directly read through the RGB channel of the smartphone. The on-site real-time detection of ZEN is realized in the linear range from 1 to 250 ng/mL, and the detection limit is as low as 0.6979 ng/mL. In the two studies, the signal amplification ability of the AuPt nanozyme composite system was applied to the actual detection by resolving the interface steric hindrance and enriching the target concentration.

### 4.3. Foodborne Pathogenic Bacteria

Pathogenic bacteria pose a major threat to global public health, causing millions of cases of foodborne diseases and a large number of deaths every year, and causing a heavy burden on personal health, medical systems and socio-economic development [81,82]. These microorganisms, especially bacteria, pollute food through a variety of ways, such as contaminated water sources (irrigation or cleaning), improper food processing (such as cross-contamination of raw and cooked food), incomplete cooking, and temperature control during storage and transportation [83,84]. In order to effectively control these hazards, rapid, sensitive and accurate detection technology is essential to monitor bacterial contamination in the entire food supply chain [85].

**Figure 5:**
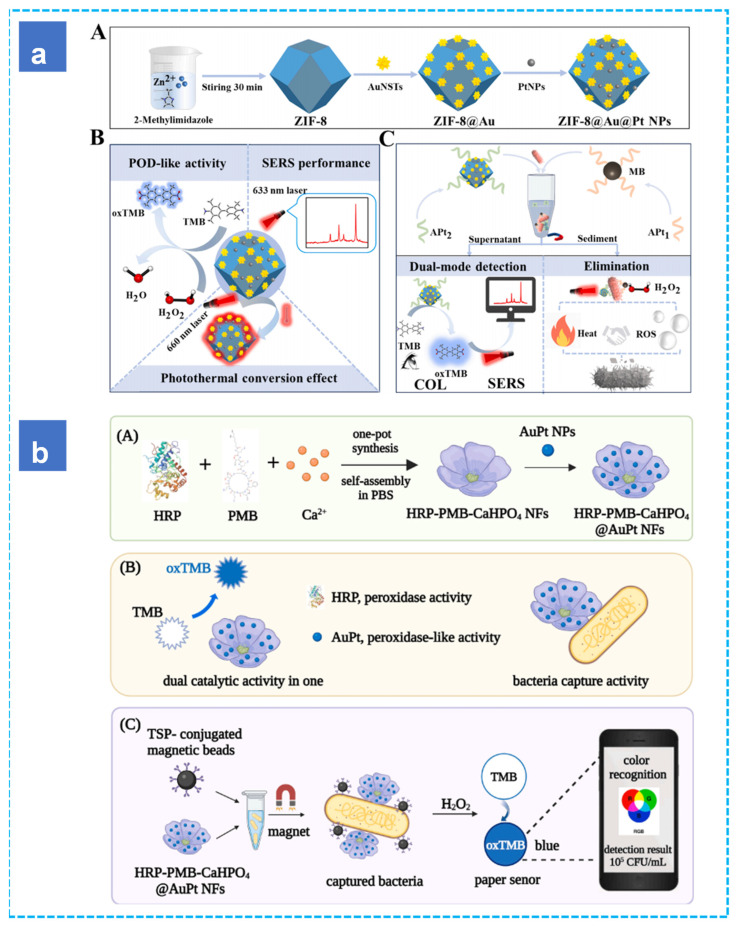
Schematic diagram of AuPt nanozyme sensor for detecting pathogenic bacteria. (**a**) Scheme of Au@Pt nanoparticles loaded onto ZIF-8 with peroxidase-like and photothermal activities for sensitive detection and efficient elimina-tion of Listeria monocytogenes. (**A**) Preparation process of multifunctional nanocomposites ZIF-8@Au@Pt NPs; (**B**) The main characteristics of nanocomposites ZIF-8@Au@Pt NPs (**C**) The process of detection and sterilization.Reprinted with permission from Ref. [87], Copyright (2024) Elsevier. (**b**) Schematic diagram of S. typhimurium detection based on dual signal amplification by four-in-one HRP-PMB-CaHPO4@AuPt nanoflowers. (**A**) Preparation of HRP-PMB-CaHPO4@AuPt nanoflowers. (**B**) The synthesized HRP-PMB-CHPO4@AuPt nanoflow-ers can both target gram-negative bacteria using the biorecognition unit (PMB) and enhance enzymatic activity by dual signal amplification elements (HRP and AuPt). (**C**) The TSP-MBs were utilized for specific separation of S. typhimurium. Reprinted with permission from Ref. [62], Copyright (2025) Elsevier.

AuPt bimetallic nanozymes can achieve precise enhancement of catalytic activity through MOF carrier confinement, hybrid nanoflower assembly, and biomimetic structure design, and can construct a multimodal sensing platform for ultrasensitive detection of pathogenic bacteria. To enhance the activity of the system, researchers used Zr/Zn-based MOFs such as PCN-224 and ZIF-8 as carriers to load AuPt particles in situ (Figure 5a) [86,87]. The high specific surface area of MOFs was utilized to inhibit component aggregation, strengthen electronic synergy, and significantly enhance peroxidase-like activity. In addition, synthesized through a one-pot method, HRP-PMB-CaHPO_4_@AuPt four-element hybrid nanoflowers integrate natural enzymes, recognition units, and nanozymes and achieve efficient signal amplification through dual catalytic unit synergy (Figure 5b) [62].

Such studies often use a magnetic capture sandwich-specific recognition system: functionalized magnetic beads are used as capture probes, and AuPt nanozyme complexes are used as signal probes. After magnetic separation, the complex formed by the target bacteria is catalyzed by TMB-H_2_O_2_ to produce a signal negatively correlated with bacterial concentration. In terms of detection performance, this type of system exhibits excellent sensitivity, with a detection limit as low as 10 CFU/mL for *Escherichia coli O157: H7* and 3.28 × 10^1^ CFU/mL for Salmonella typhimurium. In particular, the colorimetric SERS dual-mode sensing platform has an SERS detection limit of up to 5 CFU/mL for Listeria monocytogenes, effectively meeting the rapid and high-throughput screening needs of pathogenic bacteria in complex matrices such as milk and meat.

### 4.4. Food Additives

Food additives play a vital role in the modern food industry. They are widely used to improve the flavor, texture and appearance of food, extend the shelf life, and provide convenience in the processing process [88,89]. However, with the increase in processed food consumption, people are increasingly worried about the potential health risks of food additives, especially the possible harm of synthetic additives [90]. The emergence of nanozyme detection systems provides a new possibility to solve this problem. Due to its high sensitivity, specificity and rapid detection ability, it shows broad application prospects in the field of food safety detection [91].

For trace illegal additives, the high catalytic efficiency of AuPt nanozymes provides a sensitive means of signal amplification. Qi et al. investigated Au@Pt nanozyme hydrogel SERS substrate (Au@Pt@Agar); the substrate has both peroxidase-like and oxidase-like activities and excellent SERS enhancement effect [92]. Its three-dimensional network structure provides a wealth of active sites, which can catalyze the oxidation of colorless substrate TMB to blue oxide oxTMB with a strong SERS signal. Based on this, researchers have established nitrite (NO_2_^−^) sensing methods using a diazotization reaction. Under acidic conditions, NO_2_^−^ reacts with aromatic amino groups of oxTMB, resulting in a decrease in the SERS signal (1611 cm^−1^) intensity of oxTMB. The logarithm of the signal difference (ΔI) has a good linear relationship with the logarithm of the concentration with the increase in the concentration of NO_2_^−^. The linear range is from 10^−12^ to 10^−3^ M, and the detection limit is as low as 2.27 × 10^−13^ M.

In the field of meat component certification, Hendrickson et al. investigated Au@Pt. The peroxidase-like activity of the nanozyme was used as a marker to construct a highly sensitive immunochromatographic strip for the detection of pork ingredients illegally added to meat products [93]. In this study, myoglobin in porcine skeletal muscle was used as a molecular marker, and specific monoclonal antibodies were used to recognize myoglobin from pigs. By optimizing the antibody and Au@Pt coupling ratio of the nanozyme (20 μg antibody/mL Au@Pt), the best detection performance was obtained. The visual detection limit of myoglobin was 1.5 ng/mL in the conventional colorimetric mode (direct observation of the dark brown of the nanozyme itself). Following further utilization of Au@Pt, the detection limit was reduced to 0.5 ng/mL, which was about 30 times higher than that of the traditional gold nanoparticle labeled immunochromatographic method, and three times higher than that of nonamplified nanozyme colorimetry.

### 4.5. Food Freshness Detection

Food freshness monitoring is of great significance to protect public health and reduce food waste [94]. About 1.3 billion tons of food is wasted every year, one third of which is caused by food spoilage. However, traditional food freshness assessment methods often have shortcomings such as being insufficiently sensitive, time-consuming and destructive, which makes it difficult to meet the needs of real-time and non-destructive monitoring [95,96]. In response to the limitations of traditional evaluation methods, AuPt bimetallic nanomaterials provide a new tool for real-time and non-destructive monitoring of food spoilage markers due to their excellent catalytic and sensing properties.

In the establishment of sensing mode, researchers achieved a breakthrough in the detection dimension through the combination of morphology engineering and bionic technology. Zhang et al. synthesized sea-urchin-like compounds by a one-step Pt@Au nanoparticle (SU-Pt@Au NP) method [97]. Its unique spiny structure provides a wealth of catalytic active sites and shows excellent peroxidase-like activity. Based on this, the researchers constructed an indirect competitive ELISA platform and developed a dual-mode detection strategy. Under the traditional UV–vis spectrophotometer mode, the linear detection range of histamine was 0.5–100 ng/mL, and the detection limit was 0.3 ng/mL. Further, combined with the smartphone color extraction app, visual quantitative detection was realized based on the red/blue channel ratio. The linear range was extended to 0.5–1000 ng/mL, and the detection limit was as low as 0.15 ng/mL.

In order to solve the problem of poor stability of natural enzymes and biological antibodies in traditional ELISA methods, Wang et al. designed a method based on Au@Pt@Au biomimetic enzyme-linked immunosorbent assay (BELISA) based on Au complex nanozyme labeling, and an MIP biomimetic antibody was developed [98]. MIP films with specific recognition ability for histamine were synthesized in situ on 96-well plates as artificial antibodies. Meanwhile, Au@Pt@Au composite nanozymes with a three-layer core–shell structure were prepared, whose multilayer structure significantly enhanced the peroxidase-like catalytic activity. Under optimal conditions, the linear detection range of histamine was 0.01–100.00 mg/L, the detection limit was 0.069 mg/L, and the median inhibitory concentration was 7.20 mg/L. Compared with the structural analogues of 1,5-pentanediamine and β-phenylethylamine, this method showed good selectivity for histamine. The recovery rate of standard addition in yellow rice wine and liqueur was 83.80–111.00%, and there was no significant difference between the detection results of red wine and white wine samples and those of HPLC (*p* > 0.05).

A summary of Au/Pt-nanozyme-based sensors in food safety analysis is shown in Table 3.

## 5. Summary and Outlook

AuPt bimetallic nanozymes have been widely used in food safety analysis due to their tunable components, high catalytic activity, stable structure and diverse surface modifications. Firstly, chemical reduction, sol–gel, microemulsion and electrochemical deposition methods were discussed to prepare AuPt nanozymes. Next, their catalytic properties can be further regulated by optimizing external reaction conditions, including the Au/Pt atomic ratio and introducing other nanomaterials. These strategies can improve active site exposure, electron transport, target enrichment, matrix tolerance and signal amplification. Based on the above advantages, the sensor based on AuPt nanozymes has been widely used to detect veterinary drug residues, pesticide residues, heavy metal ions, mycotoxins, foodborne pathogens, food additives and freshness-related biomarkers. The reported sensing systems indicate that AuPt nanozymes can be used as a multifunctional component integrating enrichment, recognition, catalysis and signal conversion in food safety analysis.

Although AuPt nanozymes have made significant progress in laboratory-scale research, they still face many challenges for practical application. Firstly, the standardization and controllability of synthesis methods need to be further improved to obtain nanozyme materials with good batch consistency and stable catalytic performance. Secondly, the complex food matrix poses a severe challenge to the selectivity and anti-interference ability of the nanozyme sensor probe. Finally, most of the current research is still at the stage of principle verification in the laboratory. Therefore, AuPt-nanozyme-based sensing systems can be integrated with some easy strategies, such as responsive materials [99] and microfluidic platforms [100]. Specifically, the microfluidic platform can realize the integrated operation of sample introduction, enrichment, reaction and signal reading. Meanwhile, the reliability of AuPt-nanozyme-based sensing systems in actual sample detection may be improved *via* constructing standardized data sets, and introducing machine learning algorithms for background correction, feature extraction, multi-objective classification and concentration prediction. As artificial intelligence (AI) technology advances, we are confident this biosensor will continue to improve, supporting rapid clinical diagnostics. The integration of AuPt-nanozyme-based sensing systems with AI is expected to promote the development of biosensing.

## Figures and Tables

**Table 1:** Comparison table of advantages and disadvantages of different synthesis methods.

Synthetic Method	Advantage	Disadvantage
Chemical reduction	Easy operation and low cost, controllable morphology and composition ratio, suitable for large-scale production	Residual ligands or reductants
Sol–gel	Large specific surface area and high porosity, adjustable structure of gel pore	Long preparation cycle, low yield
Microemulsion	Controllable particle size, good dispersion and uniform morphology	Residual solvents and surfactants, high cost
Electrochemical deposition	Controllable structure, no chemical pollution	High requirements for substrate, difficult to prepare on a large scale, and high cost

**Table 2:** Comparison of representative kinetic parameters of Au and Pt bimetallic nanozymes.

Nanozyme Materials	Substrate	K_m_ (mM)	V_max_ (μM·s^−1^)	Ref.
Pt@Au@HP1-HP2@Fe_3_O_4_	TMB	0.4089	0.9533	[51]
	H_2_O_2_	/	/	
AuPt alloy nanozyme	TMB	0.050	0.1279	[52]
	H_2_O_2_	6.32	0.1544	
AuPt@ZIF-67	TMB	/	/	[53]
	H_2_O_2_	2.68	0.0187	
Pt-Au dendritic nanoparticles	TMB	0.22	0.282	[54]
	H_2_O_2_	/	/	
Au_2_Pt nanozyme	TMB	0.044	0.1937	[55]
	H_2_O_2_	6.12	0.2130	

**Table 3:** A summary of AuPt-nanozyme-based sensors in food safety analysis.

Detection Test Item	Detection Type	Nanomaterial	Detection Range	Limit of Detection	Ref.
Gentamicin	Colorimetry	Fe–Au@Pt	0.001–100 ng/mL	1.1 pg/mL	[68]
	Colorimetry	GO/Au-AuPt	0.00005–100 ng/mL	4.9 pg/mL	[69]
Chlorpyrifo	Fluorescence	Au@Pt	/	1.47 pg/mL	[70]
Organopho-sphorus pesticides	Colorimetry	Pt@Au	1.51–151.35 nM	0.047 nM	[57]
Hg^2+^	Colorimetry	AuPt@DSN	0.1 nM–10 μM	8.58 pM	[74]
Cr^6+^	Colorimetry	Au@Pt	0.096–38.46 μM	9.62 nM	[75]
Aflatoxin B1 (AFB1)	Colorimetry	Au@Pt/Au	0.01–1 ng/mL	/	[80]
Zearalenone (ZEN)	Colorimetry	AuPt@PIL-gel	1–250 ng/mL	0.6979 ng/mL	[58]
*Salmonella typhimurium*	Colorimetry	HRP-PMB-CaHPO@AuPt	10^2^–10^7^ CFU/mL	3.28 × 10^1^ CFU/mL	[62]
*Escherichia coli*	Colorimetry	AuPt/PCN-224	10^1^–10^6^ CFU/mL	10^1^ CFU/mL	[86]
*Listeria monocytogenes*	SERS	ZIF-8@Au@Pt NPs	10^1^–10^6^ CFU/mL	5 CFU/mL	[87]
Histamine	Colorimetry	Au@Pt@Au	0.01–100 ppm	0.069 ppm	[98]
Histamine	Colorimetry	SU-Pt@Au NPs	0.0005–1 ppm	0.15 ppb	[97]

## Data Availability

No new data were created or analyzed in this study. Data sharing is not applicable to this article.
